# 75. High Rates of Virologic Suppression with DTG/3TC in Newly Diagnosed Adults with HIV-1 Infection and Baseline Viral Load >500,000 c/mL: 48-Week Subgroup Analysis of the STAT Study

**DOI:** 10.1093/ofid/ofab466.075

**Published:** 2021-12-04

**Authors:** Charlotte-Paige M Rolle, Mezgebe Berhe, Tulika Singh, Roberto Ortiz, Anson K Wurapa, Moti Ramgopal, Dushyantha Jayaweera, Peter Leone, Jessica Matthews, Michael Cupo, Mark Underwood, Kostas Angelis, Brian Wynne, Deanna Merrill, Christopher T Nguyen, Jean A van Wyk, Andrew Zolopa

**Affiliations:** 1 Orlando Immunology Center, Orlando, Florida; 2 North Texas Infectious Diseases Consultants, Dallas, TX; 3 University of California, Riverside, Palm Springs, CA; 4 Bliss Healthcare Services, Orlando, Florida; 5 Infectious Disease Specialists of Atlanta, Atlanta, GA; 6 Midway Specialty Care Centers, Fort Pierce, Florida; 7 University of Miami, Miami, Florida; 8 ViiV Healthcare, Chapel hill, North Carolina; 9 GlaxoSmithKline, Collegeville, PA; 10 GSK, London, England, United Kingdom

## Abstract

**Background:**

The primary analysis of the STAT study demonstrated the feasibility, efficacy, and safety of using DTG/3TC as a first-line regimen in a test-and-treat setting through 24 weeks, with therapy adjustments for baseline resistance or hepatitis B virus (HBV) co-infection. Here we present secondary analyses through Week 48 of virologic outcomes in participants by baseline viral load (VL).

**Methods:**

STAT is a single-arm study of treatment-naive adults with HIV-1 infection who initiated DTG/3TC ≤ 14 days after HIV-1 diagnosis without availability of screening/baseline laboratory results. If baseline testing indicated DTG or 3TC resistance, HBV co-infection, or creatinine clearance < 30 mL/min/1.73 m^2^, then antiretroviral therapy (ART) was potentially adjusted and participants remained on study. Efficacy analyses included proportion of participants with HIV-1 RNA < 50 c/mL regardless of ART regimen at Week 48, among all participants (ITT-E missing = failure analysis) and among participants with available HIV-1 RNA data at Week 48 (observed analysis).

**Results:**

Of 131 enrolled, DTG/3TC treatment was adjusted in 10 participants, and of those with available data (n=7), all (100%) achieved HIV-1 RNA < 50 c/mL at Week 48. At Week 48, 82% (107/131) of all participants (Figure 1) and 97% (107/110) of those with available data (Figure 2) achieved HIV-1 RNA < 50 c/mL. Of participants with baseline VL ≥ 500,000 c/mL, 89% (17/19) achieved HIV-1 RNA < 50 c/mL at Week 48; the remaining 2 withdrew from study. Of participants with baseline VL ≥ 1,000,000 c/mL, 90% (9/10) achieved HIV-1 RNA < 50 c/mL at Week 48 (Table); the remaining participant withdrew consent. Of the 17 participants with baseline VL ≥ 500,000 c/mL with available data through Week 48, 76% (13/17) achieved virologic suppression by Week 24. One participant with baseline VL ≥ 500,000 c/mL switched from DTG/3TC before the Week 48 assessment. Of the 9 participants with baseline VL

≥ 1,000,000 c/mL with available data through Week 48, most participants (8/9; 89%) were suppressed by Week 24.

Figure 1. Virologic outcomes at Week 48, overall and by baseline VL and CD4+ cell count: ITT-E missing = failure analysis.

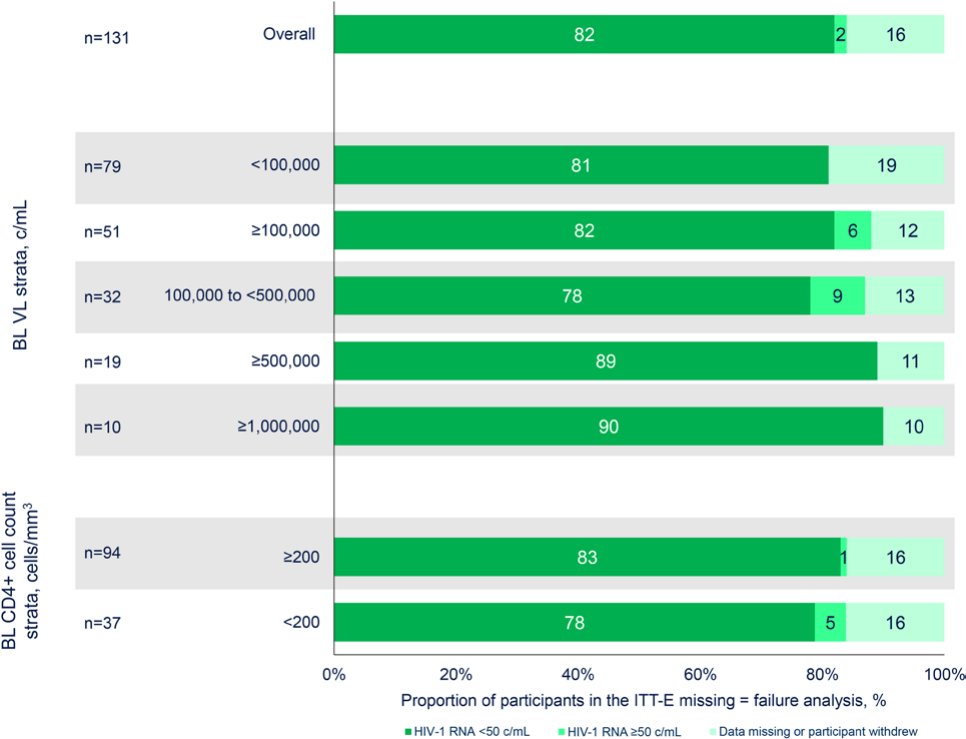

Figure 2. Virologic outcomes at Week 48, overall and by baseline VL and CD4+ cell count: observed analysis.

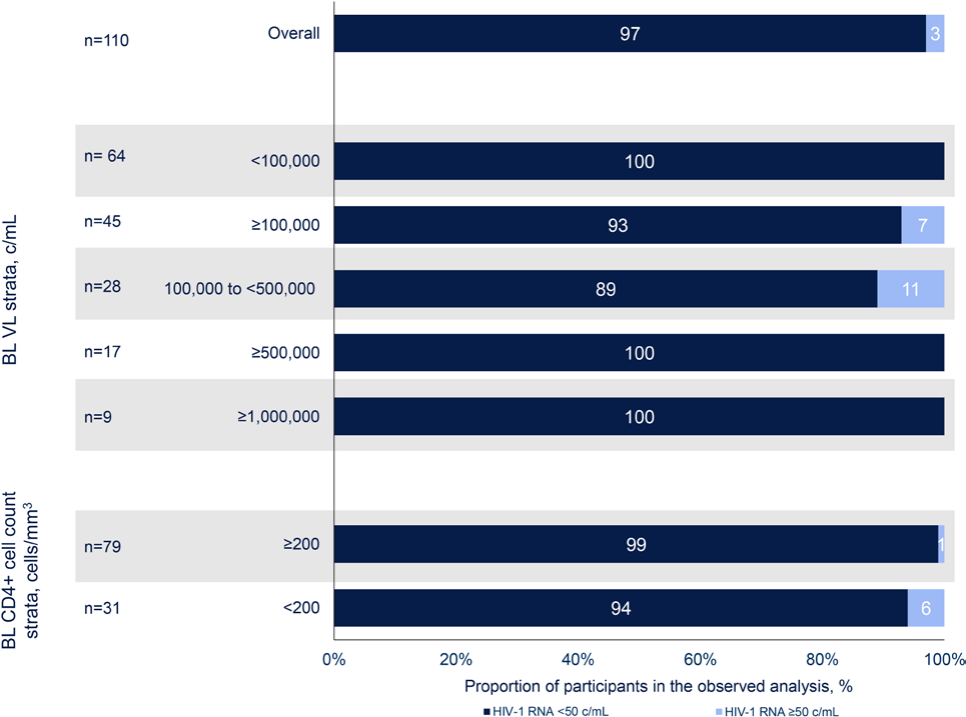

Table. Viral Load by Study Visit Among Participants with Baseline HIV-1 RNA ≥1,000,000 c/mL

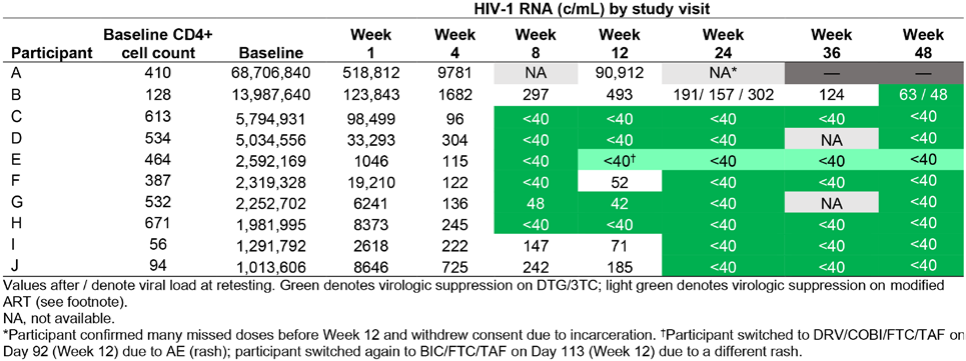

**Conclusion:**

These data provide evidence for the efficacy and feasibility of using DTG/3TC as a first-line regimen in a test-and-treat setting, including among participants with very high baseline VL.

**Disclosures:**

**Charlotte-Paige M. Rolle, MD MPH**, **Gilead Sciences** (Grant/Research Support, Scientific Research Study Investigator, Speaker’s Bureau)**Janssen Infectious Disease** (Scientific Research Study Investigator, Advisor or Review Panel member)**ViiV Healthcare** (Grant/Research Support, Scientific Research Study Investigator, Advisor or Review Panel member, Speaker's Bureau) **Tulika Singh, MD MS AAHIVS**, **Gilead** (Grant/Research Support, Advisor or Review Panel member)**ViiV** (Grant/Research Support, Advisor or Review Panel member, Speaker's Bureau) **Moti Ramgopal, MD FIDSA**, **Abbvie** (Scientific Research Study Investigator, Speaker's Bureau)**Gilead** (Consultant, Scientific Research Study Investigator, Speaker's Bureau)**Janssen** (Consultant, Scientific Research Study Investigator, Research Grant or Support, Speaker's Bureau)**Merck** (Consultant, Scientific Research Study Investigator)**ViiV** (Consultant, Scientific Research Study Investigator, Speaker's Bureau) **Dushyantha Jayaweera, MD, mrcog(uk), face**, **Gilead** (Research Grant or Support)**Janssen** (Research Grant or Support)**viiv** (Research Grant or Support) **Peter Leone, MD**, **viiv healthcare** (Employee) **Jessica Matthews, BS**, **ViiV Healthcare** (Employee) **Michael Cupo, Ph.D.**, **GlaxoSmithKline** (Employee) **Mark Underwood, PhD**, **GlaxoSmithKline** (Shareholder)**ViiV Healthcare** (Employee) **Kostas Angelis, PhD**, **GSK** (Employee, Shareholder) **Brian Wynne, MD**, **ViiV Healthcare** (Employee, Shareholder, I have shares in GSK, the part owner of ViiV) **Deanna Merrill, PharmD, MBA, AAHIVP**, **GlaxoSmithKline** (Shareholder)**ViiV Healthcare** (Employee) **Christopher T. Nguyen, MD**, **ViiV Healthcare** (Employee) **Jean A. van Wyk, MB,ChB**, **GlaxoSmithKline** (Shareholder)**ViiV Healthcare** (Employee) **Andrew Zolopa, MD**, **GlaxoSmithKline** (Shareholder)**ViiV Healthcare** (Employee)

